# Validation of the Farsi version of the Suicide Ideation and Behavior Scale

**DOI:** 10.3389/fpsyt.2023.1201193

**Published:** 2023-09-06

**Authors:** Ali Mehrabi, Azam Naghavi, Mohammad Ershad Afsharzada, Sören Friedrich, Thomas Forkmann, Heide Glaesmer, Tobias Teismann

**Affiliations:** ^1^Department of Psychology, Faculty of Education and Psychology, University of Isfahan, Isfahan, Iran; ^2^Department of Counseling, Faculty of Education and Psychology, University of Isfahan, Isfahan, Iran; ^3^Department of Psychology, Mental Health Research and Treatment Center, Ruhr-Universität Bochum, Bochum, Germany; ^4^Department of Clinical Psychology and Psychotherapy, University of Duisburg-Essen, Duisburg, Germany; ^5^Department of Medical Psychology and Medical Sociology, University of Leipzig, Leipzig, Germany

**Keywords:** suicide ideation, suicide attempts, assessment, Afghanistan, validation study

## Abstract

**Objective:**

Suicide ideation and suicide attempts are prevalent in Farsi speaking populations. The present study aimed at validating the Farsi version of the Suicide Ideation and Behavior Scale (SIBS).

**Methods:**

Reliability and validity of the Farsi version of the SIBS were established in a highly burdened Afghan student sample (*N* = 279). Internal consistency, convergent and discriminant validity were investigated, and confirmatory factor analysis was conducted.

**Results:**

The Farsi version of the SIBS was shown to have a unidimensional structure with excellent internal consistency, as well as good convergent and divergent validity.

**Discussion:**

The results suggest that the SIBS is a brief, reliable, and valid measure of current suicidal ideation and behavior that can be used in Farsi speaking populations.

## Introduction

Suicide ideation and suicidal behavior is prevalent in Iran (1, 2) and Afghanistan (3, 4). Furthermore, suicide ideation and suicide attempts are frequent in migrant populations from the respective countries (5). Suicide ideation and suicide attempts are among the strongest risk factors for suicide (6), and they are deeply distressing (7). Accordingly, it is important to have validated instruments for assessing suicide ideation and suicidal behavior for both research and clinical practice. In clinical practice, the use of questionnaires is justified in particular by the fact that affected persons regularly find it easier to disclose suicidal ideation in a questionnaire than in a personal conversation (8).

At this time, there are two validated questionnaire to assess suicidal ideation and behavior available in Farsi: the Suicidal Behavior Questionnaire-Revised [SBQ-R; (9)] and the Depressive Symptom Inventory–Suicidality Subscale [F-DSI-SS; (10)]. The SBQ-R has originally been developed by Osman et al. (11) and comprises four items that capture different facets of suicide ideation and behavior (lifetime suicidal thoughts, plans, and attempts; suicidal thoughts during the past 12 months; expression of suicidal intentions; likelihood of future suicidal acts). The Farsi version of the SBQ-R showed good internal consistency (α = 0.82) in an Iranian undergraduate sample and was shown to be positively correlated with single item assessments of suicide acceptability and lifetime suicide ideation [*r*s > 0.30; (9)]. However, the SBQ-R has been criticized for using an inconsistent item response format and mixing of different time perspectives (prospective, retrospective) within the four items (12). Furthermore, the SBQ-R does not assess current suicide ideation. The DSI-SS has originally been developed by Joiner et al. (13) and assesses with four items the frequency and intensity of suicidal ideation within the past 2 weeks. The Farsi version of the DSI-SS showed good internal consistency (α = 0.89) in an Iranian college student sample and was shown to be positively correlated with a measure of suicide-specific rumination (10). However, the DSI-SS does not capture suicidal intentions or attempts.

On this background, a frequently used German questionnaire, the Suicide Ideation and Behavior Scale [SIBS; (14), German name: Skala Suizidales Erleben und Verhalten, SSEV], was developed and translated into Farsi. The SIBS comprises nine items to measure the frequency and intensity of passive and active suicidal thoughts, suicidal intentions, suicidal impulses, suicidal plans and suicide attempts within the last 4 weeks. Lifetime suicide attempts are assessed with two additional items. Teismann et al. (14) created the SIBS based on existing guidelines for the exploration of suicide ideation and behavior (15) and under acknowledgement of current classification systems and definitions of suicidal ideation and behavior (16). The scale has a unidimensional structure and showed good internal consistency (α = 0.92) in German non-clinical and clinical adult samples. Furthermore, the scale has been shown to significantly correlate with depression, hopelessness, perceived burdensomeness as well as positive mental health. Scores in the SIBS did not differ between men and women; yet, clinical samples displayed higher scores than non-clinical samples (14). In a recent adaptation of the scale for children and adolescents (aged > 11 years), the unidimensional structure of the scale was replicated in a sample of adolescent German outpatients (17). The SIBS for Kids has a good internal consistency (α = 0.89) and shows significant correlations with symptom burden, perceived burdensomeness and thwarted belongingness; girls displayed higher scores than boys.

Taken together, it seems as if the SIBS is a reliable and valid assessment tool for suicide ideation and behavior. To enable cross-cultural research as well as clinical work in refugee populations the scale has recently been translated into various languages (Albanian, Arabic, English, French, Kurmanji, Russian, Spanish, Sorani, Tigrinya, Turkish, Ukrainian; all versions available on www.q-cultural.de). Psychometric evaluation studies on the different language versions of the SIBS are pending. On this background, the current study aimed to examine the factor structure, psychometric properties and construct validity of the Farsi version of the SIBS within an Afghan student sample. Afghan students have been shown to suffer from high levels of posttraumatic stress, depression and suicidality (3) making it a very relevant population to focus on. Furthermore, Shoib et al. (18) recently called for the adaptation of validated assessment tools for the Afghan population in order to improve psychosocial and medical care in the country.

In light of the aforementioned studies (14, 17), we expected to replicate the unidimensional structure of the SIBS in this Farsi speaking sample. Furthermore, significant positive correlations were expected between SIBS scores and depression, PTSD and suicide ideation/behavior scores; whereas a significant negative correlation was expected between positive mental health and the SIBS score. Finally, we did not expected to find gender differences in SIBS scores [cf. (3, 14)]. The present validation study was implemented as part of a larger study on psychological well-being of Afghan students (19).

## Methods

### Participants and procedure

The sample consisted of 279 university students (61.6% women; age: 18–21 years: 43%; 22–24: 36.6%; 25–27: 6.5%; 28–30: 14%) affiliated with Herat University, Eshragh University, Jami University and Ghaleb University in Afghanistan. Participants were recruited via personal invitations or invitations forwarded via social media between July and November 2022. The study invitation indicated that the survey would address suicidal ideation as well as traumatic experiences. The participation was voluntary and participants received no compensation for taking part in the study. The Ethics Committee of the Faculty of Psychology, Ruhr-Universität Bochum approved the implementation of the present study (790/2022). All participants were properly instructed and provided their informed consent to participate online.

### Measures

*Suicide Ideation and Behavior Scale* [SIBS; (14)]: The SIBS comprises six items assessing (passive/active) suicide ideation, suicidal intent, suicidal impulses and suicide planning within the last 4 weeks. Items are rated on a 6-point Likert-type scale (0 = *never*, 5 = *many times a day*). Three further items assess the occurrence of a suicide attempt within the last 4 weeks as well as occurrence/frequency of lifetime suicide attempts. The questionnaire was translated to Farsi using the back translation method (20): The English version of the SIBS was translated by a researcher from Iran, than it was reviewed by two researchers from an Afghan background. Afterwards it was back-translated to English by a different Farsi speaking researcher, who was unfamiliar with the original SIBS items. The original items and instructions of the SIBS and the back-translated items and instructions were than independently compared by two clinical researchers from Germany. There were no substantial discrepancies regarding all SIBS items. Furthermore, there were no specific issues that arised during the translation process, as such there were no words or expressions that were difficult to translate from English to Farsi [cf. (9, 10)].

*Suicide Behaviors Questionnaire-Revised* [SBQ-R; (11); Farsi version: (9)]: The SBQ-R comprises four items assessing different facets of suicidal ideation and behavior (lifetime suicide ideation, suicide plans, suicide attempts, 12-month suicide ideation, suicidal communication, and one’s estimation of how likely a future suicide attempt might be). Each item utilizes a different Likert scale with a sum score of 18 points indicating the highest severity of suicidal risk. Internal consistency was good (*α* = 0.79) in the current sample. At the time the present study was conducted, the SBQ-R was the only validated suicide questionnaire available in Farsi and was therefore chosen to establish convergent validity.

*PTSD Checklist* [PCL-5; (21); Farsi version: (22)]: The PCL-5 assesses symptoms of PTSD (e.g., intrusion, avoidance, cognitive and mood alteration, arousal) with 20 items (e.g., “In the past month, how much have you been bothered by: Having strong negative feelings such as fear, horror, anger, guilt, or shame?”) that are rated on a 5-point Likert-type scale (0 = *not at all*, 4 = *extremely*). The higher the sum score, the higher the symptoms of PTSD. Internal consistency was *α* = 0.91 in the current sample.

*Patient Health Questionnaire* [PHQ-9; (23); Farsi version: (24)]: The Patient Health Questionnaire (PHQ-9) assesses symptoms of depression (e.g., lack of interest, feeling down, poor appetite) with nine items (e.g., “Over the last 2 weeks, how often have you been bothered by any of the following problems: Little interest or pleasure in doing things?”) that are rated on a 4-point Likert-type scale (0 = *not at all*, 3 = *nearly every day*). Internal consistency was *α* = 0.88 in the current sample.

*Positive Mental Health Scale* [PMH-Scale; (25); Farsi version: (26)]: The PMH-Scale assesses subjective and psychological aspects of well-being across nine items (e.g., “I manage well to fulfill my needs”; “I feel that I am actually well equipped to deal with life and its difficulties”) that are rated on a 4-point Likert-scale (0 = *do not agree*, 3 = *agree*). Higher scores indicate higher levels of PMH. Internal consistency was *α* = 0.85 in the current sample.

### Statistical analyses

IBM SPSS Statistics Version 27 (27) and R (version 4.1.3, Package:lavaan) were used for statistical analysis. Participants who scored higher than 0 in the SIBS were classified as suffering from suicidal ideation [cf. (28)]. Participants who affirmed the SIBS item on lifetime suicide attempts (item 8) were classified as lifetime suicide attempters. Distribution of the data was verified using the Kolmogorov–Smirnov and Shapiro–Wilk tests. Subsequently, the means, standard deviations as well as skewness and kurtosis of all items, as well as their influence on the internal consistency of the scale were analyzed. In order to check the fit of the one-factorial model determined by Teismann et al. (14), a confirmatory factor analysis (CFA) based on items 1 to 6 was carried out. Due to the ordinal scale level of the SIBS, the Robust Weighted Least Square Estimator (WLSM) was used. The following fit indices were used to assess the model fit: Fit Index χ^2^ according to Satorra-Bentler-correction (29), the Root Mean-Square Error of Approximation (RMSEA) with 90% confidence interval (90% CI), the Comparative-Fit-Index (CFI), the Tucker-Lewis Index (TLI) and the standardized Root Mean Square Residual (SRMR). The cut-off values were interpreted according to the guidelines of Hu and Bentler (30): RMSEA values of <0.05 indicate a good model fit, values between <0.08 and >0.05 indicate an adequate model fit (31). Values of >0.90 for the CFI and the TLI and for the SMRM values of <0.09 indicate good model fit (30). Next, to investigate the validity of the SIBS, its associations with positive mental health, as well as with depressive symptoms, PTSD and suicide ideation/behavior were assessed by the calculation of zero-order bivariate correlation analyses. Group differences in SIBS scores between participants with or without lifetime suicide attempts were tested using a *t*-test. Gender differences regarding suicidal ideation and lifetime suicide attempts were tested using a *t*-test and a Chi-Square Test.

## Results

Mean scores and standard deviations of all questionnaires are displayed in Table 1. In total 44.4% of the participants reported suicidal ideation within the past 4 weeks (SIBS > 0). Thirty-six participants (12.9%) reported a lifetime suicide attempt.

**Table 1:** Descriptive statistics of the investigated variables (*N* = 279).

	*M (SD)*	*Min–Max*
PCL	36.60 (15.83)	2–80
PHQ-9	10.58 (6.46)	0–27
SIBS	2.83 (5.06)	0–30
SBQ-R	5.61 (3.51)	0–18
PMH	16.76 (5.78)	0–30

*N* = 279; PMH, Positive Mental Health Scale; PCL-5, PTSD Checklist; PHQ-9, Patient Health Questionnaire; PMH-Scale, Positive Mental Health Scale; SIBS, Suicide Ideation and Behavior Scale; SBQ-R, Suicide Behaviors Questionnaire-Revised; *M*, Mean; *SD*, Standard deviation; *Min*, Minimum; *Max*, Maximum.

### Item analysis

The results of the Kolmogorov–Smirnov as well as the Shapiro–Wilk-test showed that the participants answers were not normally distributed (*p* < 0.001). These results were confirmed by the item analysis (see Table 2). Selectivity is good for all items (>0.3).

**Table 2:** Results of the item analysis of the SIBS (*N* = 279).

Item during the past 4 weeks	M	SD	Selectivity	Kurtosis	Skewness	α, if item is deleted
1. I thought it would be better if I wasn’t alive.	0.81	1.35	0.57	2.57	1.86	0.88
2. I thought about killing myself.	0.50	1.02	0.80	6.10	2.46	0.83
3. I seriously considered killing myself.	0.34	0.87	0.83	11.06	3.19	0.83
4. I intended to kill myself.	0.35	0.85	0.80	11.75	3.22	0.84
5. I have had the urge to kill myself.	0.52	1.23	0.64	21.32	3.83	0.86
6. I have planned exactly how I will kill myself.	0.33	1.04	0.58	44.46	5.62	0.87

SIBS, Suicide Ideation and Behavior Scale.

### Confirmatory factor analysis and internal consistency

The one factorial model determined from the exploratory factor analysis (EFA) and subsequent principal component analysis (PCA) by Teismann et al. (14) was tested using confirmatory factor analysis (CFA). The results of the confirmatory factor analysis indicate a good global model fit: Satorra-Bentler corrected χ^2^ = 19.481 (df = 8, *p* = 0.012), CFI = 0.989, TLI = 0.980, RMSEA = 0.072 (90%-CI = 0.031–0.113) and SRMR = 0.025. Local fit was good as well, as all factor loadings were >0.60 (see Table 3).

**Table 3:** Factor loadings of the SIBS items (*N* = 279).

Item during the past 4 weeks	SIBS
1. I thought it would be better if I wasn’t alive.	0.69
2. I thought about killing myself.	0.87
3. I seriously considered killing myself.	0.91
4. I intended to kill myself.	0.89
5. I have had the urge to kill myself.	0.76
6. I have planned exactly how I will kill myself.	0.70

SIBS, Suicide Ideation and Behavior Scale.

Internal consistency was assessed using Cronbach’s *α*. In the current study, it was *α* = 0.875.

### Construct validity

The SIBS was positively correlated with depression (*r* = 0.404, *p* ≤ 0.000), posttraumatic stress disorder symptoms (*r* = 0.390, *p* ≤ 0.000) and suicide ideation/behavior as assessed with the SBQ-R (*r* = 0.654, *p* ≤ 0.000). The SIBS was negatively correlated with positive mental health (*r* = −0.430, *p* ≤ 0.000). Men and women did neither differ in suicide ideation, *t*(277) = −0.397, *p* = 0.692, nor in lifetime suicide attempts, *χ*^2^ = 0.44, df = 1, *p* = 0.51. Participants with lifetime suicide attempts (*n* = 36) suffered from higher levels of concurrent suicidal ideation as measured by the SIBS (*M* = 10.583, *SD* = 7.911) than participants without lifetime suicide attempts (*n* = 243; *M* = 1.687, *SD* = 3.191), *t*(277) = −12.152, *p* ≤ 0.000.

## Discussion

In the present study, the reliability and construct validity as well as the postulated factor structure of the Farsi version of the Suicide Ideation and Behavior Scale (SIBS) were investigated. In line with the original version of the SIBS (14) and the version for children and adolescents (17), the Farsi version of the SIBS had a unidimensional factor structure and an excellent internal consistency. Construct validity of the SIBS was supported by expected associations between the SIBS and depressive symptoms, posttraumatic stress symptoms, suicide ideation/behavior as assessed with the SBQ-R and positive mental health. SIBS suicide ideation scores (items 1 to 6) differentiated between participants with vs. without lifetime suicide attempts. In line with previous investigations (3, 14), men and women did not differ in suicide ideation scores in the current study. Taken together, findings indicate that the Farsi version of the SIBS is a reliable and valid instrument. The SIBS also meets the following evaluation criteria—established by Batterham et al. (32)—for assessing suicidal ideation and suicidal behavior: (1) It explicitly captures suicidal ideation and suicidal behavior, (2) it is brief and easy to complete, (3) it collects quantitative data, and 4. it is available free of charge.^1^

However, it should be highlighted that the SIBS only assesses current suicidal ideation and behavior; with the exception of lifetime suicide attempts. Other instruments must therefore be used if information on suicidal ideation over different time periods has to be assessed. The SIBS and SBQ-R complement each other rather well in this regard and may be used in conjunction. It should also be noted that the SIBS—just as any other assessment instrument—can help to make an assessment of the severity and nature of suicide ideation and behavior; it cannot be used to predict suicidal behavior and/or suicide death or to be seen as a marker of immediate suicide risk (33, 34). In general questionnaires should only ever be used as a supplement to a more detailed conversation about suicidal ideation and behavior.

Several limitations have to be considered when interpreting the current results. First, since 100% of the sample were Afghan students, it is unclear how the findings would generalize to a more diverse Farsi speaking population and to clinical populations. Yet, with regard to suicide ideation/behavior, student populations are a group of special concern (35). Moreover, the proportion of those who affirmed suicidal ideation and suicide attempts in the present study was as large as otherwise found in clinical samples (36). It is possible that a differentiation between clinical and non-clinical samples in highly stressed societies—with low access to mental health facilities—such as Afghanistan is therefore less meaningful. Sadly, it must be emphasized that due to the fact that the Taliban banned female students from universities in December 2022 (37), a study of Afghan female students will hardly be possible in future studies. Second, the cross-sectional design of the current study precludes analyses of test–retest reliability over short periods of time. Longitudinal studies on this issue are warranted. Third, the current study utilized only self-report measures. This method has certain advantages, for example, the measures are economical and easy to administer. However, self-report measures may fail to capture suicide ideation/behavior, depressive symptoms, or post-traumatic symptoms in their full complexity. Fourth, to keep the assessment as confidential as possible [cf. (38)], only gender and age group were assessed to characterize the sample. Therefore, it is not possible to give more details on the sample composition. Finally, the questionnaire can only be used by Farsi (Persian) speakers in Iran and Afghanistan (or Persian speaking minorities in Tajikistan or Uzbekistan). The scale has not been validated for use with Afghans who speak Dari or regional dialects.

## Conclusion

Despite these limitations, the current results suggest that the Farsi version of the SIBS is a brief, reliable, and valid measure of concurrent suicidal ideation and behavior that can be used to complement mental health assessments in research and practice. With regard to the use of the questionnaire, it should finally be emphasized that questionnaires cannot be used meaningfully for the prediction of suicidal behavior (33), that answering questions about suicidality does not instill or intensify suicidality in respondents (39), but that nevertheless—in the context of studies on suicide or suicide-associated constructs—participants must always be provided with emergency contact information (40). The latter represents a real challenge in countries such as Afghanistan, where help for mental health problems is in general only inadequately available (41).

## Data availability statement

The raw data supporting the conclusions of this article will be made available by the authors, without undue reservation.

## Ethics statement

The studies involving humans were approved by Ethics Committee of the Faculty of Psychology, Ruhr-Universität Bochum, Germany. The studies were conducted in accordance with the local legislation and institutional requirements. The participants provided their written informed consent to participate in this study.

## Author contributions

AM, AN, MA, SF, TF, HG, and TT contributed to the conception and design of the study as well as the interpretation of the data. MA conducted the acquisition of data. TF, SF, HG, and TT conducted the data analysis. AM, AN, and TT drafted a first version of the article and finalized the revised version. All authors contributed to the article and approved the submitted version.

## Conflict of interest

The authors declare that the research was conducted in the absence of any commercial or financial relationships that could be construed as a potential conflict of interest.

## Publisher’s note

All claims expressed in this article are solely those of the authors and do not necessarily represent those of their affiliated organizations, or those of the publisher, the editors and the reviewers. Any product that may be evaluated in this article, or claim that may be made by its manufacturer, is not guaranteed or endorsed by the publisher.

## Footnotes

^1^ www.q-cultural.de
